# The unknown story of an early intraluminal inferior vena cava filter prototype

**DOI:** 10.1016/j.jvsv.2024.101940

**Published:** 2024-06-28

**Authors:** Oscar Moreno, Alexis Roth, Enrico Ascher, Anil P. Hingorani

**Affiliations:** a Section of Vascular Surgery, Department of Surgery, University of Michigan, Ann Arbor, MI; b State University of New York, Downstate Health Sciences University, College of Medicine, Brooklyn, NY; c Department of Surgery, Total Vascular Care, Brooklyn, NY; d Department of Surgery, NYU Langone Hospital—Brooklyn, Brooklyn, NY

Among the worldwide recognized innovators that contributed to the development of inferior vena cava (IVC) filter prototypes in the 1960s by Kazi Mobbin-Uddin (1967; umbrella IVC filter) and Lazar J. Greenfield (1973; cone-shaped IVC filter), and earlier open surgical techniques including sutures by Marion DeWeese (1958; grid-harp filter with silk sutures), and external devices as IVC clips by James A. and James T. DeWeese (1965; Adams-DeWeese clip),^1^, ^2^, ^3^, ^4^ there is one unknown name, Bertram D. Cohn (1919-2002), who developed a previously unknown intraluminal IVC filter prototype.^5^ This development was significant because it proposed a novel intraluminal device different from most of the other open surgical techniques requiring retroperitoneal exposure of the IVC and placed as external devices.^1^^,^^2^

Cohn was a pediatric surgeon based in Brooklyn, New York, who in 1964 developed a previously unknown intraluminal caval filter consisting of a three-tiered conical shape device with expanding wings made in stainless steel or vitallium alloy. Cohn's IVC filter prototype was tested in 1965, submitted as an abstract to the American College of Surgeons in 1966, and patented in 1967 before Mobbin-Uddin published his results (Fig 1). He first performed experimental work in dogs at the Maimonides Medical Center Hospital in Brooklyn, New York, developing an animal model for the occlusion of the IVC through a saphenous or external jugular venotomy. This model allowed him to test a multiprolonged spring occlusion device, obtaining pathological evidence of the trapped thrombi after harvesting the implanted devices from his survival animal model.

**Fig 1:**
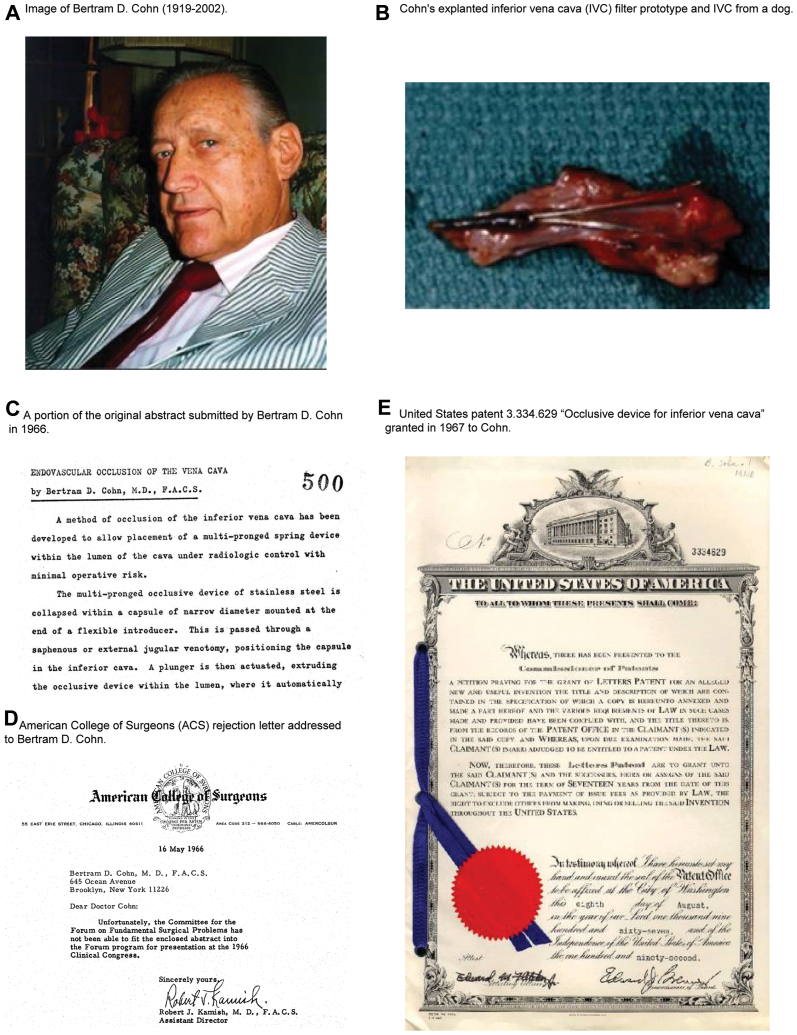
**(A)** Image of Bertram D. Cohn (1919-2002). **(B)** Cohn's explanted inferior vena cava (IVC) filter prototype and IVC from a dog. **(C)** A portion of the original abstract submitted by Bertram D. Cohn in 1966. **(D)** American College of Surgeons rejection letter addressed to Bertram D. Cohn. **(E)** United States patent 3.334.629 "Occlusive device for inferior vena cava" granted in 1967 to Cohn.^5^

Nevertheless, when Cohn attempted to present his creation, his abstract was rejected by the 1966 American College of Surgeons forum program. It is impossible to know the exact reasons for the abstract rejection. Still, it might be related to the limited abstracts accepted owing to a highly exclusive and competitive abstract applications at the time from multiple surgical fields. During the 1960s, kidney and liver transplants, coronary artery bypass surgery using the saphenous vein, and new interventional radiology techniques were first achieved. Also, it is expected to find barriers to novel ideas including medical devices. The diffusion of innovations theory dictates that any idea, practice, or object requires individual adopters (who are likely to make the adjustments needed to adopt the proposed changes) to diffuse the proposed innovation successfully. This theory was described first in 1962 by Everett Rogers (1931-2004) and is commonly used in industry and markets to understand the adoption of novel technology.^6^

In 1967, Cohn obtained his US patent for this experimental device, which described the use of local anesthetics, fluoroscopy or other radiological control, minimal operative risks, and consisted of inserting a flexible introducer percutaneously containing the collapsed encapsulated occlusion device. After positioning the capsule in the IVC, the plunger (cable with push rod) was actuated, extruding the occlusive device in the lumen.^6^ All parts of his occlusive device were patented, including the multiprolonged spring device, cylindrical introducer, and push rod (Fig 2). The additional required parts of his novel occlusion device were made of gum rubber (for the resilient collar to prevent the entry of the blood in the tube), polyethylene, or polyvinyl chloride acetate copolymer for the cylinder, offering relatively inert materials which could be sterilized. For the cable or push rod, a stiffer material was required, such as polyethylene, nylon, or stainless steel wire offering a better compressive strength to eject the occlusion device from the cylinder.^6^

**Fig 2:**
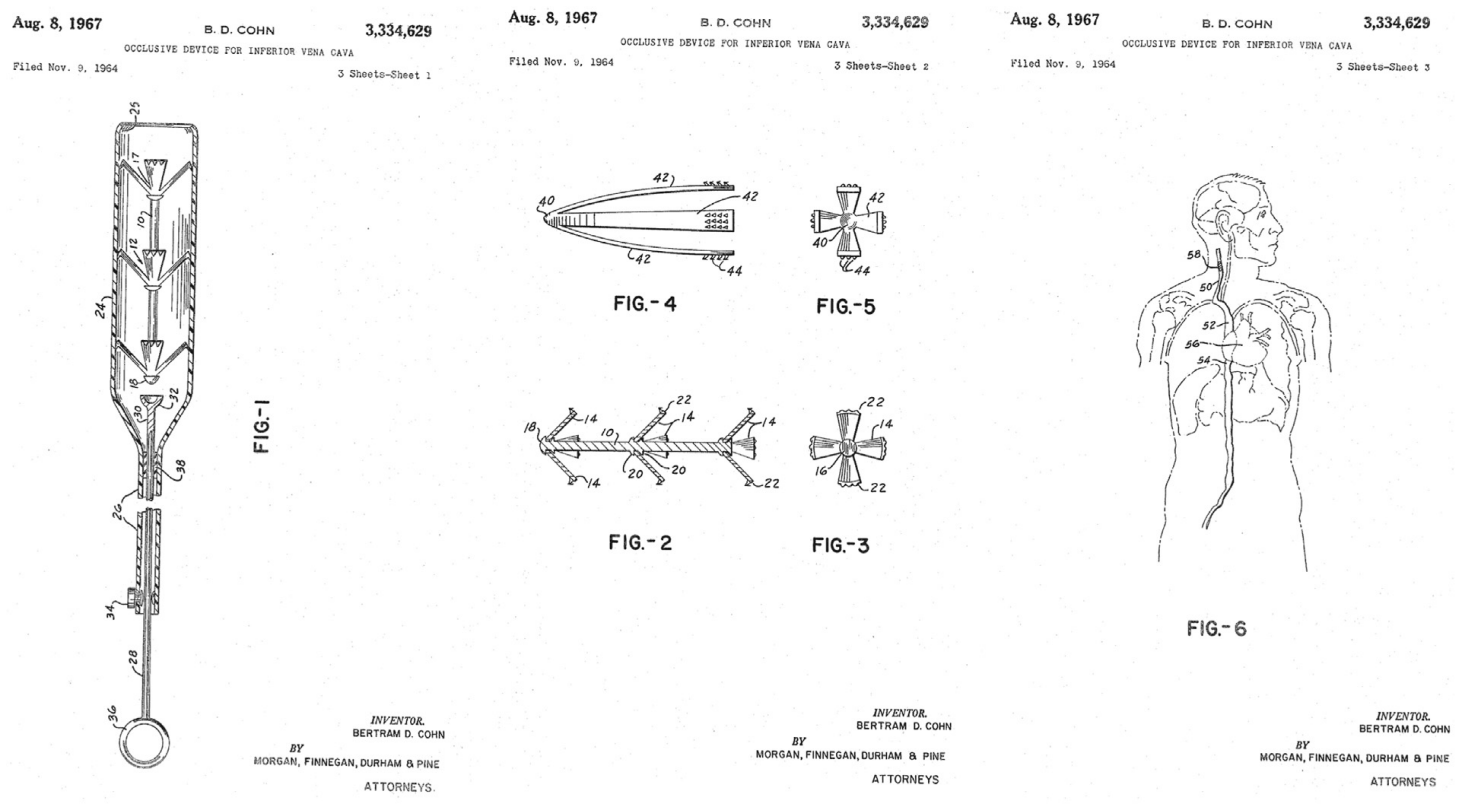
The 1967 Cohn United States images on the patent 3.334.629 "Occlusive device for inferior vena cava" showing the multiple patented parts and detailed implantation method.^6^

Bertram Cohn successfully developed a model to cause pulmonary embolization in dogs and tested a novel IVC medical device. Initially, the academic pitfalls for diffusing his idea did not stop him from patenting his discovery afterward. However, he did not continue pursuing further attempts to validate his IVC filter prototype in animal or human clinical trials to further develop his prototype. His unknown effort had the potential to revolutionize vascular surgery by preventing pulmonary embolisms and inspire others to build on his shoulders and push a novel idea until it was accepted. It is essential to recognize and promote the traits among innovators' personalities, where passion, grit, resilience, and perseverance are commonly key factors to pursue an idea and overcome obstacles and challenges, driving people to achieve and generations of inspired innovators.^7^ We need to promote innovation and research to find novel and better solutions, building teams and leaders to tackle our field's past, present, and future limitations in vascular surgery.

## Disclosures

None.
